# ZnO Modulates Swine Gut Microbiota and Improves Growth Performance of Nursery Pigs When Combined with Peptide Cocktail

**DOI:** 10.3390/microorganisms8020146

**Published:** 2020-01-21

**Authors:** Xiaoyuan Wei, Tsungcheng Tsai, Joshua Knapp, Kristopher Bottoms, Feilong Deng, Robert Story, Charles Maxwell, Jiangchao Zhao

**Affiliations:** 1 Department of Animal Science, Division of Agriculture, University of Arkansas, Fayetteville, AR 72701, USA; 2 Farm Animal Genetic Resources Exploration and Innovation Key Laboratory of Sichuan Province, Sichuan Agricultural University, Chengdu 611130, China

**Keywords:** nursery pigs, gut microbiota, growth performance, peptide cocktail, ZnO

## Abstract

Zinc has been very efficacious in reducing post-weaning diarrhea, whereas animal-derived peptides are suggested to improve the growth performance of weaned piglets. However, the combined effect of zinc and peptides on swine production and swine gut microbiota is still largely unknown. In this study, we followed 288 nursery pigs from the age of d30 to d60 to evaluate the growth performance and gut microbiota of weanling pigs subjected to different levels of a fish-porcine-microbial peptide cocktail (0.05%, 0.25%, and 0.5%) with or without the pharmaceutical level of zinc oxide (ZnO) (2500 ppm) supplementation in a nutrient-deficient diet. Rectal swab samples were collected from pigs with body weight (BW) approach average at each pen on d30, d42, and d60 to determine gut microbiota. Average daily gain (ADG) and BW in piglets fed high zinc (HZ) increased with increasing levels of peptide. The microbiota of the HZ group also diverged from those of the standard zinc (SZ) group from d30 to d60. Adding peptide did not alter community structure regardless of zinc supplementation. Collectively, these findings demonstrated that the pharmaceutical level of zinc as ZnO conditioned the gut community to the point where peptide could effectively restore growth performance in nursery pigs fed nutrient-deficient diets.

## 1. Introduction

Weaning is one of the most stressful events in a pig’s life due to drastic environmental and nutritional changes. Newly weaned pigs are faced with both psychosocial and physical stressors including maternal separation, abrupt changes in diet, transportation, co-mingling, and establishment of new social hierarchy, etc. [1,2]. Weaning stress could induce dysfunction of the intestinal barrier, which is characterized by increased intestinal permeability [3,4]. This intestinal dysintegrity allows pathogens, antigens, and endotoxins to “leak” into the body, resulting in systemic inflammation and disease [5,6,7]. In addition, weaning can disturb the ecological balance of the gut microbial community which contributes to the animal’s health through a variety of mechanisms such as the activation of immune response, competition with pathogens for nutrients, bacteriocin production, and acid environmental maintenance, etc. [8,9]. Because weaning negatively affects piglet growth and overall health, it becomes necessary to stimulate the indigenous gastrointestinal microflora and maintain their balance because of the protection they offer against the invasion of pathogenic bacteria [10]. 

Zinc is an essential mineral and micronutrient for humans and animals. It is involved in a multitude of body functions, ranging from acid–base balance to immunocompetency, and as an activator or co-factor of many enzymes. Optimizing zinc in diet can prevent infectious diseases [11,12] and reduce the incidence of diarrhea and respiratory tract infections in young children [13,14]. In pig production, bioavailable dietary zinc at 125 ppm is sufficient to maintain proper physiological functions [15]. The pharmaceutical level of zinc has been used to reduce morbidity and mortality in the swine industry [16,17]. 

Zinc oxide (ZnO), an inorganic compound of zinc, at the pharmaceutical level is an alternative feed medication to control post-weaning diarrhea in pig. Pharmacological concentrations of ZnO effectively suppress the incidence of post-weaning diarrhea [18] through its ability to induce oxidative stress by producing reactive oxygen species (ROS), which results in bacterial death [19]. Wang and colleagues reported that high ZnO supplementation suppressed the growth of *Escherichia coli* and coliforms and left the abundance of *Lactobacilli* and *Clostridium XIVa* unaffected [20]. However, animal studies have yielded inconsistent effects of ZnO on intestinal bacteria. For example, Højberg et al. found a reduced number of lactic acid bacteria and an increased number of coliforms, whereas others observed that *E. coli* was unaffected, accompanied by reductions in anaerobic and lactic acid bacteria [21,22]. When a weaning piglet adapts to a solid diet, the small intestinal villi undergo a short period of villus atrophy and crypt hyperplasia. ZnO has been shown to downregulate the inflammatory gene expression [23,24], thereby alleviating weanling-induced intestinal injury [25,26,27].

Peptide, a high quality protein derived from food animals’ hydrolysate, can be rapidly absorbed in the intestine and also have many beneficial effects besides meeting nutrition requirements, such as anti-inflammatory and antioxidant capabilities [28,29]. Hydrolysates of porcine origin, spray-dried porcine intestine or mucosa, have been shown to improve growth performance of post-weaning piglets [30,31] and increase body weight of broilers on day 22 after the hatchery [32]. Another study on feeding broilers with peptide derived from Atlantic salmon viscera showed that they had superior growth performance compared with fishmeal- or plant protein-fed groups [33]. In addition, feeding fish with fish protein hydrolysates could improve their growth performance, immune status, and disease resistance, and enhance feed utilization and protein digestibility [34,35,36]. Thus, hydrolyzed animal proteins, as feed supplements, are significant in their ability to increase animal productivity.

The proposed health benefits of probiotics are associated with improving gut health, enhancing the immune system and the bioavailability of nutrients, and decreasing lactose intolerance symptoms [37]. Probiotic-derived peptides are possible antibacterial agents. For instance, *Lactobacillus rhamnosus* (LR 231) produces a number of antimicrobial peptides that can eliminate human pathogens and food spoilage organisms, such as *Pseudomonas aeruginosa*, *Enterobacter aerogenes*, *Escherichia coli*, *Staphylococcus aureus*, *Bacillus cereus*, *Salmonella* spp., *Campylobacter jejuni*, *Helicobacter pylori*, *Listeria monocytogenes*, and *Bacillus megaterium* [38,39]. Lu et al. also identified two small peptides produced by *Lactobacillus* GG (LGG) that exerted bactericidal activity against *E. coli* EAEC 042, *S. typhi*, and *S. aureus* [40]. 

Feed cost represents about 70% of the total production cost in the swine industry. Therefore, increasing the nutrient utilization efficiency not only increases production profit but also reduces excess nutrient excretion, which minimizes the detrimental effects on the environment. Peptides are a cost preferable alternative when compared to high-dollar protein ingredients, such as spray-dried porcine plasma. In this study, we formulated nutrient-deficient diets by removing fish meal and reducing soybean meal in nursery phases (NP) 2 and 3, respectively, to determine if the cost-effective peptides could replace the expensive proteins in the diet while still sustaining a comparable growth performance in pigs. Due to the public concern regarding antibiotic resistance and environmental contamination, feed additive medication and high-level zinc will soon be phased out in livestock production. Alternative feed additives to optimize the weaning herd’s growth performance are greatly needed. The objective of this study was to determine whether or not peptides alone or in combination with a high-zinc diet alter fecal microbiota and improve growth performance. If the answer is yes, then this study will provide a modulation target of the swine gut microbiota that might replace high-level zinc and improve swine production.

## 2. Materials and Method

The pigs were cared for following the guideline of the University of Arkansas’s Institutional Animal Care and Use Committee (IACUC# 17082 was issued on 22 May 2017).

### 2.1. Animal and Diets

Upon weaning (21 d of age), a total of 288 weaned pigs (PIC C-29 × 380) from the University of Arkansas’s Swine Research Unit were transferred to a nursery facility where they were blocked by initial body weight and then allotted into 1 of 8 pens within blocks (*n* = 6). An attempt was made to balance sex within blocks so that each treatment was represented by equal numbers of each sex within the block. Pigs remained in the same pens throughout the experiment. A total of 6 pigs were housed in each pen (1.5 × 1.2 m^2^), and each pen was equipped with a two-hole feeder and one waterer for ad libitum access to diets and water. Due to low feed consumption and complications during early weaning, nutrient requirements for early weaning pigs are not well defined. Therefore, all pigs were fed a common phase 1 diet (crude protein: 22.8%, standardized ileal digestible (SID) lysine: 1.46%) and inorganic Zn from ZnSO_4_, which was supplemented in a trace mineral premix to meet the nutrient requirement of zinc (195 ppm of zinc in complete diets). At the end of phase 1, pens were randomly assigned to one of eight dietary treatments: (1) Negative control (NC): standard zinc nutrient-deficient diet; (2) P0.05: NC + 0.05% peptide; (3) P0.25: NC + 0.25% peptide; (4) P0.5: NC + 0.5% peptide; (5) Positive control (PC): high-zinc nutrient-sufficient diet; (6) PZ0.05: P0.05 + ZnO; (7) PZ0.25: P0.25 + ZnO; (8) PZ0.5: P0.5 + ZnO (Table S1, Supplementary Materials). Peptide used in the trial was a fish-porcine-microbial peptide mixture (Peptiva, Vitech Bio-Chem Corporation, Glendale, CA, USA). Based on the manufacturer’s recommendation, peptide concentration of 0.5% is recommended for their customers. To test whether or not a further reduction in the dose of peptide could have similar benefits on improving nutrient utilization, 0.25% and 0.05% concentrations of peptide were arranged. Nutrient composition of the peptide mixture is presented in Table S2, Supplementary Materials. Positive control (PC) diets were formulated to meet the nutrient requirements suggested for swine (2012) [41], whereas fish meal (nursery phase 2) and soybean meal (nursery phase 3) were reduced to lower dietary crude protein at 1.6% (−0.1% SID lysine) to create a negative control (NC) treatment. To evaluate Zn concentration effects, we further grouped NC, P0.05, P0.25, and P0.5 into a standard zinc group (SZ, 195 ppm Zn), and PC, PZ0.05, PZ0.25, and PZ0.5 into a high-zinc group (HZ, 2500 ppm of Zn). Weaning pigs were allowed to acclimatize themselves to the nursery facility for 10 days and a 30-day feeding trial followed. Pigs were fed a common phase 1 diet and then switched to experimental diets (Table S3, Supplementary Materials) for phases 2 and 3 (nursery phase NP1: d21–30; NP2: d31–42; NP3: d43–60). Individual piglet body weight (BW) was measured at the end of the adaptation period (d30), and individual pig BW and pen feed disappearance were recorded at each subsequent phase change to determine the average daily gain (ADG), average daily feed intake (ADFI), and feed efficiency (G:F). Blood samples (*n* = 8/subgroup) were collected into K2EDTA tubes via jugular vena cava from the median BW piglet of each pen at the end of each phase to determine complete blood count (CBC) using a Hemavet 950 (Drew Scientific, Miami Lakes, FL, USA).

### 2.2. Gut Microbiota Analyses

Fecal swab (Puritan Opti-Swab, Puritan Medical Products, Gulford, ME, USA) samples (*n* = 8/subgroup) were collected from the same piglets used for blood collection on d30, d42, and d60 and were stored at −80 °C before being subjected to DNA extraction. 

DNA was extracted from 200 μL of sample using the DNeasy PowerLyzer PowerSoil Kit (Qiagen, Germantown, MD, USA) according to the manufacturer’s protocol. DNA concentration was measured by a NanoDrop One (Thermo Fisher Scientific, Madison, WI, USA) and diluted to 10 ng/μL. 16S rRNA gene libraries were constructed as described previously [42,43]. Briefly, the V4 region of 16S rRNA from each sample was amplified using the forward primer (5′-GTGCCAGCMGCCGCGGTAA-3′) and reverse primer (5′-GGACTACHVGGGTWTCTAAT-3′) with attaching Illumina sequencing primer and barcode sequence. The PCR amplicons were pooled together in equimolar concentrations using the SequalPrep Normalization Plate Kit (Invitrogen, Carlsbad, CA, USA). Library concentration was determined by qPCR using the Kappa Library Quantification Kit (Roche, Indianapolis, IN, USA) with primers specific to the Illumina adapters. The quality of the library was determined by an Agilent 2100 Bioanalyzer (Agilent, Santa Clara, CA, USA). The pooled library was then sequenced on an Illumina MiSeq sequencer with paired end (2 × 250 bp, MiSeq Reagent Kit v2, 500 cycles (Illumina, San Diego, CA, USA)). A commercial community DNA was included as a positive control (ZymoBIOMICS™ Microbial Community Standard (Zymo Research, Irvine, CA, USA)). Negative controls from DNA extraction and PCR amplification were also sequenced for quality controls.

Sequencing reads were analyzed using mothur v1.39.5 [44] following the MiSeq SOP, including steps for quality-filtering, alignment against a 16S reference database (SILVA v132), and clustering into operational taxonomic units (OTUs) with a 97% identity threshold. The OTUs were then classified against the RDP (Ribosomal Database Project) database. Unlike growth performance data where the pen was used as the experimental unit, an individual pig was used as the experimental unit for microbiome data analysis. The gut microbial diversity within each subgroup and the distances between subjects were evaluated by alpha-diversity (Shannon index, Observed OTUs) and beta-diversity (Bray–Curtis, Jaccard) measures, respectively. ANOSIM (analysis of similarity) was performed to evaluate the dissimilarity between groups (or subgroups) by using mothur v1.39.5. LEfSe analysis was used to identify specific bacteria that were enriched in each group (or subgroup) at the OTU level (https://huttenhower.sph.harvard.edu/galaxy/). 

### 2.3. Growth Performance Data Analysis

Growth performance and CBC data were analyzed by a mixed effects regression model using the Mixed procedure of SAS 9.3 (SAS Institute, Inc., Cary, NC, USA) as randomly complete block design [45]. Dietary treatments were the only fixed effect, blocks based on initial BW were the random effect, and each pen served as the experimental unit for ANOVA. Post hoc Tukey’s honest significance difference test was used for pairwise comparison among the groups’ least square means. To determine the effect of various levels of peptide and Zn, data from pigs fed 0.05% peptide, 0.25% peptide and 0.5% peptide or 0.05% peptide + ZnO, 0.25% peptide + ZnO, and 0.5% peptide + ZnO were further analyzed using a 2 × 3 factorial arrangement with two levels of Zn and three levels of peptide. The levels of peptide in the diets were used in the interactive matrix language (IML) procedure of SAS to generate coefficients [46], which were then incorporated into an orthogonal contrast analysis to determine increasing levels of peptide and interaction of increment peptide at various levels of Zn. 

## 3. Results

### 3.1. Growth Performance

**Figure 1:**
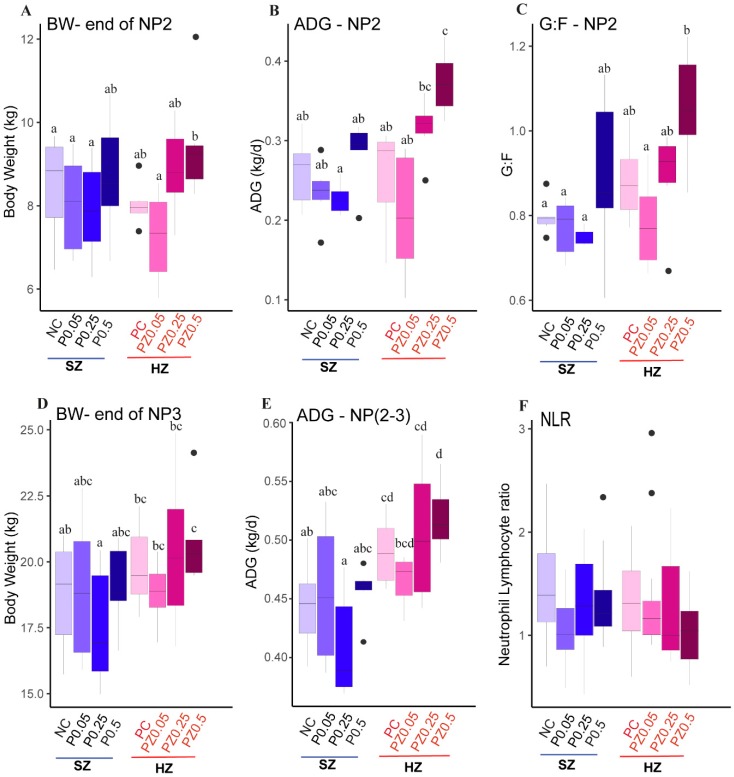
Effects of different doses of peptide with standard level of zinc (standard zinc (SZ)) and pharmaceutical level of zinc (high zinc (HZ)) on (**A**) body weight (BW, kg), (**B**) average daily gain (ADG, kg/d), (**C**) feed efficiency (G:F) of pigs during nursery phase 2 (NP2) (d31–42), (**D**) BW, (**E**) average daily gain (ADG, kg/d) during NP2 and NP3 (d31–60), and (**F**) neutrophil-to-lymphocyte ratio (NLR). At weaning (21 d), pigs were allowed to acclimatize themselves to the new environment for 10 d where a common phase 1 diet was provided. At the end of phase 1, pens were randomly assigned to one of eight dietary treatments: (1) Negative control (NC): standard zinc nutrient-deficient diet; (2) P0.05: NC + 0.05% peptide; (3) P0.25: NC + 0.25% peptide; (4) P0.5: NC + 0.5% peptide; (5) Positive control (PC): high-zinc nutrient-sufficient diet; (6) PZ0.05: P0.05 + ZnO; (7) PZ0.25: P0.25 + ZnO; (8) PZ0.5: P0.5 + ZnO (Table S1). ^a–d^ Bars with different superscripts differ significantly at *p* ≤ 0.05; outliers are displayed as black dots.

Body weight (BW, Figure 1A) and average daily gain (ADG, Figure 1B) in nursery phase 2 increased linearly with increasing levels of the peptide in the high-zinc group, whereas little response was observed in pigs fed 0.25% and 0.5% peptide in standard zinc diets (Zinc*linear peptide *p* = 0.013 and *p* = 0.018, respectively). Pigs fed PZ0.5 had superior ADG than pigs fed NC in phase 2 (Figure 1B) and this is also observed when considering the entire experimental period (phases 2 and 3, Figure 1E, Treatment, *p* < 0.0001). The peptide dose response in the HZ group led to a similar final BW to that of PC-fed pigs, and pigs fed PZ0.5 were 1.94 kg heavier than pigs fed NC diets (Figure 1D). In addition, peptide supplementation at the highest level (0.5%) in the SZ group and 0.25% and 0.5% in the HZ group had similar feed efficiency when compared to PC-fed pigs in nursery phase 2 (Figure 1C). Neutrophil-to-lymphocyte ratio (NLR) decreased with increasing levels of peptide in the HZ group, whereas a linear increased NLR was observed in the SZ group (Figure 1F, Zinc*linear peptide *p* = 0.05). In addition, NLR was negatively correlated with the ADG at the end of phase 2 in the HZ group but was not in the SZ group (HZ group: R = −0.4, *p* = 0.05; SZ group: R = −0.14, *p* = 0.5; Pearson). The current results indicate that the combination of ZnO (2500 ppm) and peptide, especially above 0.25%, had a synergetic effect on restoring growth performance of pigs fed on a nutrient-deficient diet compared to that of PC-fed pigs. This might be attributed to the decrease of systemic inflammation. The detailed data on growth performance are summarized in Tables S4–S6. 

### 3.2. Effects of Peptide Plus ZnO on Alpha and Beta Diversity

**Figure 2:**
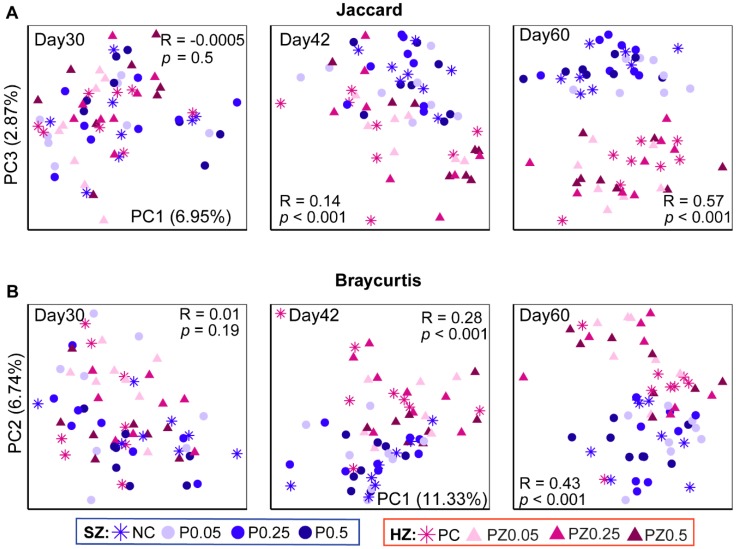
ZnO difference in gut microbiota between the SZ and HZ groups. Principal coordinates analysis (PCoA) based on (**A**) Jaccard and (**B**) Bray–Curtis distance shows patterns of separation by ZnO, irrespective of peptide concentration. The analysis of similarity (ANOSIM) procedure was used to test for significance of clustering pattern between the SZ and HZ groups.

The microbiota diversity (Shannon index, Figure S1A, Supplementary Materials) and richness (Observed OTUs, Figure S1B) were not significantly influenced by the different dietary concentrations of peptide in either the SZ or HZ group during the study period, except for that fact that the P0.5 group presented higher richness than the PC group at d60. The gut microbiota profiles of SZ and HZ piglets were considerably distinct. Community membership visualized using principal coordinates analysis (PCoA) based on the Jaccard distance (Figure 2A) revealed a gradual and significant segregation by ZnO over time (d30, R = −0.0005, *p* = 0.5; d42, R = 0.14, *p* < 0.001; d60, R = 0.57, *p* < 0.001; ANOSIM test). Consistently, PCoA based on the Bray–Curtis dissimilarities confirmed the effect of ZnO on the swine gut microbiota (d30, R = 0.01, *p* = 0.19; d42, R = 0.28, *p* < 0.001; d60: R = 0.43, *p* < 0.001; ANOSIM test) (Figure 2B). The levels of peptide in the diets did not alter microbiota structure within the HZ or SZ groups (Table S7). 

### 3.3. Effects of Peptide Plus ZnO on Gut Microbiota Composition

**Figure 3:**
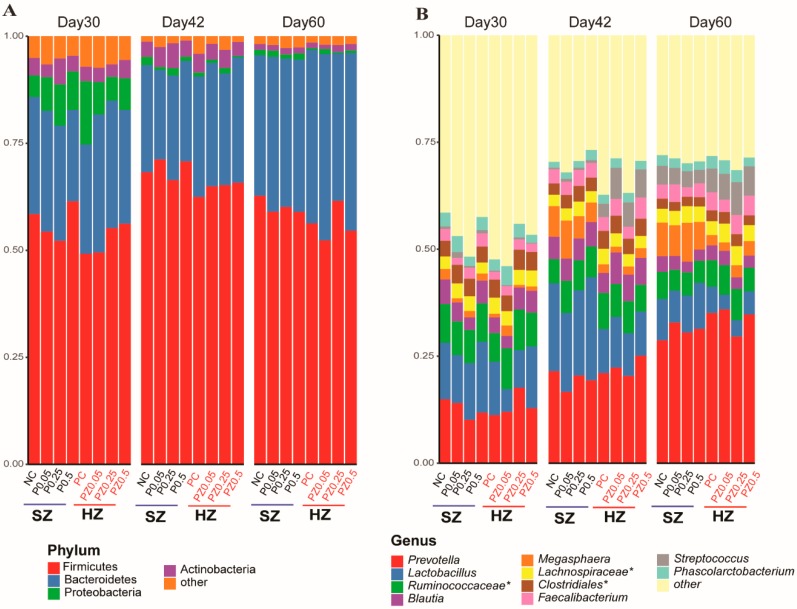
Bacterial relative abundance of each treatment. Relative abundance of (**A**) top 4 phyla and (**B**) top 10 genus-classified rectal microbiota at d30 (starting point), d42, and d60 is reported. * denotes unclassified operational taxonomic unit (OTU) reported at higher taxonomic level.

The fecal samples from the SZ and HZ groups showed many notable differences in gut microbiota composition over time. The dominant bacterial phyla were *Firmicutes* and *Bacteroidetes* followed by *Proteobacteria* and *Actinobacteria* at each phase for both groups. These four phyla accounted for 93%–98% of all sequences (Figure 3A). At the genus level, there was a significant compositional difference between piglets that received peptide only and those fed peptides plus high level of zinc (Figure 3B), which was consistent with the pattern that we observed in the gut microbiota structure (Figure 2). The pigs fed high-zinc diets lowered the relative abundance of *Lactobacillus* (d30: 10% vs. 13%; d42: 11% vs. 21%; d60: 5% vs. 9%) and *Megasphaera* (d30: 1.2% vs. 1.6%; d42: 1.9% vs. 6.4%; d60: 2.6% vs. 7.7%) compared to the SZ group throughout the study period. In addition, the relative abundance of *Streptococcus* was significantly promoted by the HZ diet compared to the SZ diet at d42; however, this difference was reduced as the piglets aged (d30: 0.3% vs. 0.8%; d42: 5.7% vs. 0.6%; d60: 6.5% vs. 3.8%).

### 3.4. Linear Discriminant Analysis of Gut Bacteria

**Figure 4:**
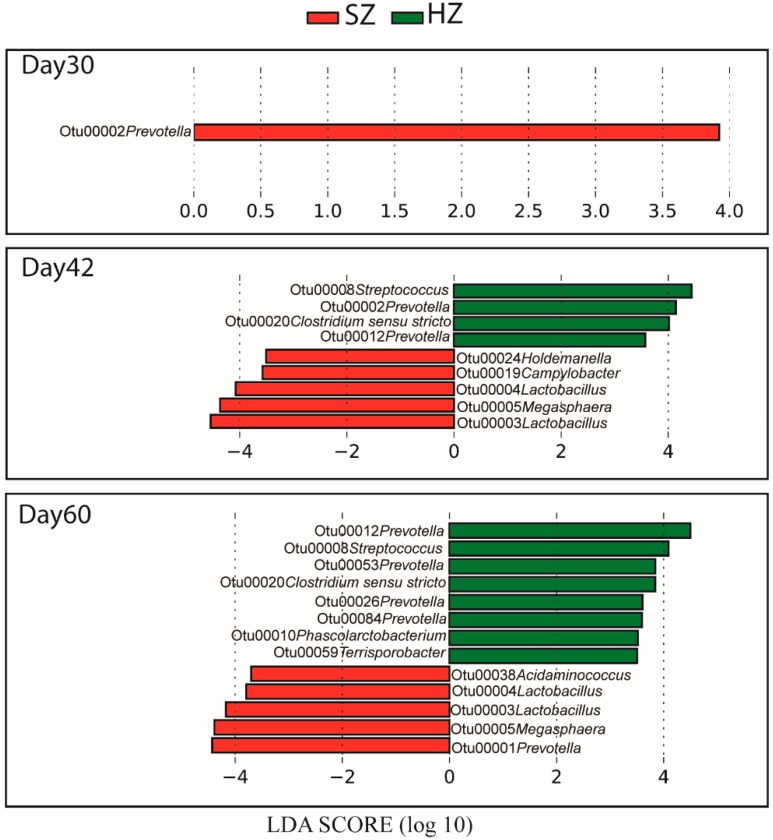
LEfSe analyses of the swine gut microbiota data. LEfSe identified significantly different bacterial taxa (at the OTU level) between the SZ and HZ groups. OTUs in this graph were statistically significant (*p* < 0.05) and had an LDA Score >3.5, which was considered a significant effect size.

**Figure 5:**
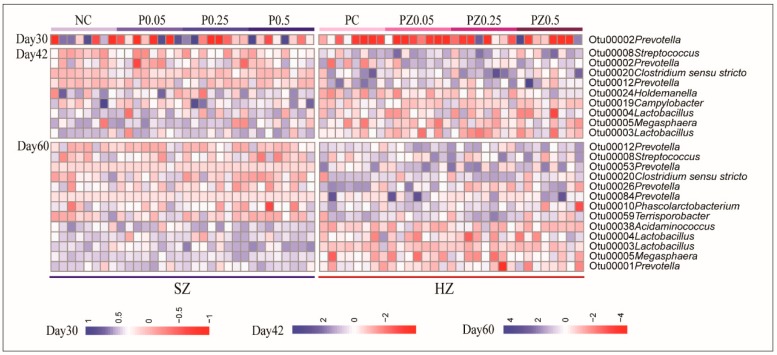
Heatmap shows the relative abundance of OTUs (log 10 transformed). Each column represents one animal and each row represents one OTU differentially represented between groups according to LEfSe results. The color intensity scale shows the relative abundance of OTU (log 10 transformed); blue denotes a high relative abundance of OTU, whereas red denotes a low relative abundance of OTU.

At the community level, our data indicates that high-zinc diet caused microbiota change during the nursery phase. We next performed LEfSe analysis to compare the gut microbiota between the HZ and SZ groups at the OTU level (Figure 4). At the beginning (d30), only *Prevotella* (OTU2) was significantly different between the HZ and SZ groups. On d42, several bacterial taxa were enriched in the HZ group; these included *Streptococcus* (OTU8), *Prevotella* (OTU2, OTU12), and *Clostridium sensu stricto* (OTU20), whereas the SZ group had significantly more abundant *Lactobacillus* (OTU3, OTU4), *Megasphaera* (OTU5), *Campylobacter* (OTU19), and *Holdemanella* (OTU24). On d60, samples from the HZ group had higher relative abundances of *Prevotella* (OTU12, OTU53, OTU26, and OTU84), *Streptococcus* (OTU8), *Clostridium sensu stricto* (OTU20), *Phascolarctobacterium* (OTU10), and *Terrisporobacter* (OTU59) compared to the SZ group, which had higher abundances of *Prevotella* (OTU1), *Megasphaera* (OTU5), *Lactobacillus* (OTU3, OTU4), and *Acidaminococcus* (OTU38). The abundances of these OTUs for individual piglets were visualized on a heatmap (Figure 5).

**Figure 6:**
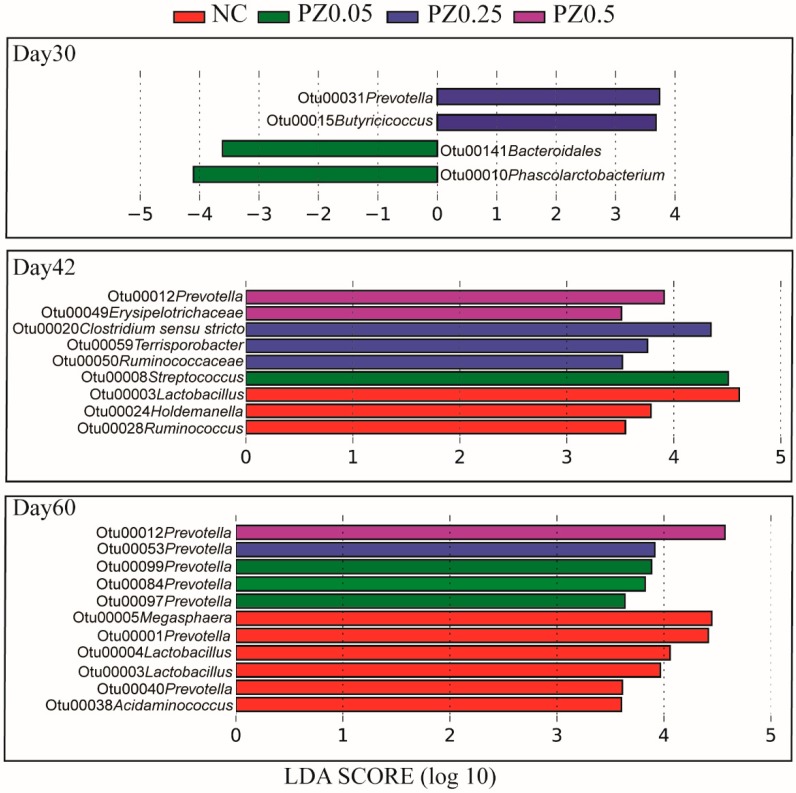
LEfSe analyses of gut bacteria in response to different levels of peptide with high level of ZnO. LEfSe identified significantly different bacterial taxa (at OTU level) among subgroups. OTUs in this graph were statistically significant (*p* < 0.05) and had an LDA Score >3.5.

In an effort to better understand how the peptide supplement at different concentrations affected gut bacteria, specific bacteria associated with different levels of peptide with or without ZnO were explored by LEfSe. The PZ0.5 subgroup samples, which displayed the best growth performance, were compared to the other subgroups (NC, PZ0.05, and PZ0.25) at d30, d42, and d60 (Figure 6). *Prevotella* (OTU12) was greater in the PZ0.5 subgroup relative to the other subgroups at both d42 and d60. Comparisons were also made between the subgroups (NC, P0.05, P0.25, and P0.5) within the SZ group at d30, 42, and 60. As expected, the results revealed that there were insignificant differences among NC, P0.05, P0.25, and P0.5 subgroups, except for OTU180 which was higher in P0.5 subgroup at d60 (Figure S2, Supplementary Materials). 

## 4. Discussion

In this study, we described the impacts of different concentrations of peptide compound (derived from porcine, fish, and probiotic) supplementation with standard-level (195 ppm) or high-level zinc (2500 ppm) on the growth performance and intestinal microbiota profiles of weanling piglets. Fecal swab samples were used to represent swine gut microbiota. Although not ideal due to the biogeography variation of swine gut microbiota [47], fecal rectal swabs are the best samples to represent the gut microbiome for longitudinal studies to follow the same sets of animals without sacrificing the animals. Our results indicated that the peptide and high-level ZnO combination restored the performance parameters of the weanling piglets fed nutrient-deficient diets, particularly at the highest peptide concentration (0.5%) level, whereas peptide supplementation at each concentration with standard-level zinc failed to produce any improvement. Our LEfSe results are consistent with the growth performance that there were insignificant differences among the subgroups within the standard zinc (SZ) group after peptide intervention. On the other hand, peptide with high-level zinc caused significant changes in growth performance and composition of the gut microbiota. Based on the lack of growth-promoting effects of ZnO on germ-free animals [48,49], we hypothesized that the beneficial effects of peptide + ZnO supplementation might be partly due to the distinguishable modulatory effect of ZnO on the gut microbiota and its metabolic regulatory function [50]. 

Neutrophil-to-lymphocyte ratio (NLR) has recently been suggested as a biological marker of systemic inflammation in many diseases [51]. Neutrophils not only promote inflammation but also maintain the whole-body inflammatory state [52]. Lymphocytes are responsible for regulating the immune system pathway [53]. NLR has been identified as a predictive and prognostic value in many human diseases [53,54,55,56]. A recent study showed that anxiety levels were positively associated with NLR in humans [57]. A study in China reported that gastric cancer patients with anxiety/depression had significantly higher preoperative NLR than those from a non-anxiety/depression group [58]. A positive correlation between NLR and stress score was also shown in patients with multiple sclerosis, a disease of autoimmunity and inflammation [59]. Note that this ratio should not be used as a sole source to assess systematic inflammation or diagnose diseases without additional supporting evidence. In our study, the NLR value decreased with increasing levels of peptide and correlated negatively with ADG in the HZ group. This may indicate that the peptide, in combination with the pharmacological concentration (2500 ppm) of ZnO, helped regulate the immune system and stress levels of pigs. This allowed pigs to distribute dietary nutrients to weight gain rather than proinflammatory response.

High-level ZnO resulted in significant changes in specific genera and OTUs. On the genus level, we observed that the relative abundance of *Streptococcus* significantly increased in piglets given high-level ZnO treatment. It has been reported that *Streptococcus* spp. have the capacity to produce serotonin, which is an important neuromodulator in the neural processing of anxiety and fear [60,61]. Thus, the increased presence of *Streptococcus* in the HZ group may help alleviate stress leading to improved growth performance of weaned piglets. Interestingly, bacterial features associated with *Streptococcus* were also identified as growth-promoting bacteria in pigs in two recent studies [43,62]. Additionally, a low gut relative abundance of *Streptococcus* was tied to low body weight piglets compared to a high relative abundance found in those of normal body weight [63]. This genus was found in high abundance as a commensal throughout the healthy human gastrointestinal tract. As a high-ranking commensal member of the gut, along with other members, their presence is very beneficial to the host. Such benefits include the following: (1) production of essential mucosal nutrients; (2) help with regulating healthy intestinal structure; (3) competitive exclusion of pathogenic bacteria along with producing antipathogenic compounds; (4) education of immune system; and (5) fermentation of dietary fiber into essential short-chain fatty acids [64]. Some species of *Streptococcus* such as *S. thermophiles* and *S. salivarius* are used as probiotics and provide some of the beneficial effects mentioned above [65,66,67].

The relative abundances of *Lactobacillus* and *Megasphaera* in the pharmaceutical levels of ZnO-treated piglets were significantly lower than those found in piglets receiving the standard zinc diet. *Lactobacillus*, an important member of the gut microbiota, is commonly used as a probiotic in the swine industry because of its beneficial health effects on the host. In this study, our data showed that the relative abundance of *Lactobacillus* rapidly decreased after feeding the piglets with the pharmaceutical level of ZnO. In agreement with this, in vitro studies have shown that *Lactobacillus acidophilus* was impeded by ZnO nanoparticles (NPs) [68]. Some in vivo studies have also demonstrated that certain *Lactobacillus* species were reduced by dietary zinc in weaned piglets and chickens [50,69]. It may be that ZnO inhibited the *Lactobacillus* directly or promoted the growth of an antagonistic species against *Lactobacillus* within the gastrointestinal tract. Interestingly, *Lactobacillus* spp. in the chicken ileum could also be reduced by antibiotic growth promoters such as tylosin [70].

*Megasphaera* is a lactate-utilizing butyrate producer [71]. It has been reported that *M. elsdenii* could effectively prevent diarrhea that is associated with hyper-lactate accumulation in pigs [72]. A hyperlactate mouse model confirmed that *M. elsdenii* is capable of restoring cecal fermentation to a normal status by reducing lactate and increasing butyrate [73]. The presence of butyrate helps improve large intestine function. It plays an important role in promoting the growth of epithelial cells, stimulating mucus release, and absorbing minerals and water [74,75,76,77,78]. In a previous study, *Megasphaera* spp. were more abundant in the stomach or ileum of a high dietary zinc group (2425 mg/kg) [79]. However, we detected a relative decrease in the concentration of *Megasphaera* in the HZ group. This difference in the two studies could be the result of a combination of several possibilities. The first possibility concerns the important time points in our experiment, which included weaning day, beginning, ending, and sampling points. For example, the weaning day in our study was day 21, whereas the other study was day 26. The second possibility could be the difference in the experimental diets. We used a combination of ZnO and peptide, whereas Starke et al. used only ZnO. The third possibility involves the difference in sample collection methods. We elected to use a swab method to collect fecal samples, whereas they collected digesta from the stomach or ileum. The microbial population changes in density and composition along the gastrointestinal tract [47,80]. Thus, analysis of samples taken from different locations in the gut could vary significantly. Finally, there is the possibility regarding the microbial exposure difference on the piglets between the housing facilities of each study that could, at least in part, explain this inconsistency. *Lactobacillus* and *Megasphaera* are usually considered beneficial gut bacteria; however, their presence decreased in the HZ group, which also had an increase in growth performance. To explain this, it may be that these bacteria established at a level of symbiotic equilibrium with the rest of the community, which provided a more efficient microbiota and resulted in a growth performance increase of nursery piglets.

In summary, the addition of ZnO (2500 ppm) to a nursery diet shaped the gut microbiota and, in combination with the highest level of the peptide (0.5%), restored growth performance when moderate nutrient-deficient diets were fed. Future studies are highly desired to isolate these bacteria altered by ZnO and to feed them to the pigs to test whether or not they have growth-promoting functions. Our peptide mixture at different concentrations supplemented with a standard zinc diet had a negligible effect on the growth performance and microbiota composition. On the other hand, as the peptide concentration increased in the presence of high levels of ZnO, so did the growth performance, which may be due to the reduction of stress or systemic inflammation based on the decreasing level of the neutrophil-to-lymphocyte ratio observed. This study may provide new insights into swine diet applications involving the combination of peptides and ZnO. Due to environmental concerns caused by the presence of Zn in pig manure in the environment and the possible development of antibiotic resistance associated with the pharmacological addition of ZnO in post-weaning piglets, further experimentation is necessary to find an effective replacement such as organic acids and probiotics. 

## Supplementary Materials

The following are available online at https://www.mdpi.com/2076-2607/8/2/146/s1, Figure S1: Alpha diversity measures for different dietary supplements at three time points, Figure S2: LEfSe analyses of gut bacteria in response to different levels of peptide in SZ group, Table S1: Dietary treatments, Table S2: Peptiva nutrient composition, Table S3: Diet ingredient, Table S4: Effect of Peptide cocktail on BW and ADG in nursery pigs fed diets with or without ZnO (least square means ± SE), Table S5: Effect of Peptide cocktail on ADFI and G:F in nursery pigs fed diets with or without ZnO(least square means ± SE), Table S6: Contrast result of BW and ADG (covariance included), Table S7: Analysis of similarity (ANOSIM) was used to study swine gut microbiome dissimilarities between the different levels of peptide within SZ or HZ group based on the Braycurtis or Jaccard distances.

## References

[1] Forbes J.M. (2007). Voluntary Food Intake and Diet Selection in Farm Animals.

[2] Whittemore C., Green D.J. (2001). Growth of the Young Weaned Pig.

[3] Boudry G., Péron V., Le Huërou-Luron I., Lallès J.P., Sève B. (2004). Weaning induces both transient and long-lasting modifications of absorptive, secretory, and barrier properties of piglet intestine. J. Nutr..

[4] Spreeuwenberg M., Verdonk J., Gaskins H., Verstegen M.J.T.J. (2001). Small intestine epithelial barrier function is compromised in pigs with low feed intake at weaning. J. Nutr..

[5] Berkes J., Viswanathan V., Savkovic S., Hecht G.J.G. (2003). Intestinal epithelial responses to enteric pathogens: Effects on the tight junction barrier, ion transport, and inflammation. Gut.

[6] Britton E., McLaughlin J.T. (2013). Ageing and the gut. Proceed. Nutr. Soc..

[7] Soeters P.B., Luyer M.D., Greve J.W.M., Buurman W.A., Care M. (2007). The significance of bowel permeability. Curr. Opin. Clin. Nutr. Metab. Care.

[8] Modesto M., D’Aimmo M.R., Stefanini I., Trevisi P., De Filippi S., Casini L., Mazzoni M., Bosi P., Biavati B.J.L.S. (2009). A novel strategy to select Bifidobacterium strains and prebiotics as natural growth promoters in newly weaned pigs. Livest. Sci..

[9] Konstantinov S.R., Awati A.A., Williams B.A., Miller B.G., Jones P., Stokes C.R., Akkermans A.D., Smidt H., De Vos W.M. (2006). Post-natal development of the porcine microbiota composition and activities. Environ. Microbiol..

[10] Vondruskova H., Slamova R., Trckova M., Zraly Z., Pavlik I. (2010). Alternatives to antibiotic growth promoters in prevention of diarrhoea in weaned piglets: A review. Vet. Med..

[11] Shankar A.H., Prasad A.S. (1998). Zinc and immune function: The biological basis of altered resistance to infection. Am. J. Clin. Nutr..

[12] Prasad A.S. (1991). Discovery of human zinc deficiency and studies in an experimental human model. Am. J. Clin. Nutr..

[13] Aggarwal R., Sentz J., Miller M.A. (2007). Role of zinc administration in prevention of childhood diarrhea and respiratory illnesses: A meta-analysis. Pediatrics.

[14] Bhan M.K., Bhandari N. (1998). The role of zinc and vitamin A in persistent diarrhea among infants and young children. J. Pediatr. Gastroenterol. Nutr..

[15] Hahn J.D., Baker D.H. (1993). Growth and plasma zinc responses of young pigs fed pharmacologic levels of zinc. J. Anim. Sci..

[16] Pettigrew J. (2006). Reduced use of antibiotic growth promoters in diets fed to weanling pigs: Dietary tools, part 1. Anim. Biotechnol..

[17] Smith J., Tokach M., Goodband R., Nelssen J., Richert B.T. (1997). Effects of the interrelationship between zinc oxide and copper sulfate on growth performance of early-weaned pigs. J. Anim. Sci..

[18] Poulsen H.D. (1998). Zinc and copper as feed additives, growth factors or unwanted environmental factors. J. Anim. Feed Sci..

[19] Dwivedi S., Wahab R., Khan F., Mishra Y.K., Musarrat J., Al-Khedhairy A.A. (2014). Reactive oxygen species mediated bacterial biofilm inhibition via zinc oxide nanoparticles and their statistical determination. PLoS ONE.

[20] Wang W., Van Noten N., Degroote J., Romeo A., Vermeir P., Michiels J. (2019). Effect of zinc oxide sources and dosages on gut microbiota and integrity of weaned piglets. J. Anim. Physiol. Anim. Nutr..

[21] Broom L., Miller H., Kerr K., Knapp J. (2006). Effects of zinc oxide and Enterococcus faecium SF68 dietary supplementation on the performance, intestinal microbiota and immune status of weaned piglets. Res. Vet. Sci..

[22] Højberg O., Canibe N., Poulsen H.D., Hedemann M.S., Jensen B.B. (2005). Influence of dietary zinc oxide and copper sulfate on the gastrointestinal ecosystem in newly weaned piglets. Appl. Environ. Microbiol..

[23] Hu C.H., Song Z.H., Xiao K., Song J., Jiao L.F., Ke Y.L. (2014). Zinc oxide influences intestinal integrity, the expressions of genes associated with inflammation and TLR4-myeloid differentiation factor 88 signaling pathways in weanling pigs. Innate Immun..

[24] Ou D., Li D., Cao Y., Li X., Yin J., Qiao S., Wu G. (2007). Dietary supplementation with zinc oxide decreases expression of the stem cell factor in the small intestine of weanling pigs. J. Nutr. Biochem..

[25] Hu C., Song J., Li Y., Luan Z., Zhu K. (2013). Diosmectite-zinc oxide composite improves intestinal barrier function, modulates expression of pro-inflammatory cytokines and tight junction protein in early weaned pigs. Br. J. Nutr..

[26] Hu C., Xiao K., Song J., Luan Z.S. (2013). Effects of zinc oxide supported on zeolite on growth performance, intestinal microflora and permeability, and cytokines expression of weaned pigs. Anim. Feed Sci. Technol..

[27] Roselli M., Finamore A., Garaguso I., Britti M.S., Mengheri E. (2003). Zinc oxide protects cultured enterocytes from the damage induced by Escherichia coli. J. Nutr..

[28] Chakrabarti S., Jahandideh F., Wu J. (2014). Food-derived bioactive peptides on inflammation and oxidative stress. BioMed Res. Int..

[29] Fitzgerald A., Rai P., Marchbank T., Taylor G., Ghosh S., Ritz B., Playford R.J.G. (2005). Reparative properties of a commercial fish protein hydrolysate preparation. Gut.

[30] Zimmerman D. (1996). Interaction of Intestinal Hydrolysate and Spray-Dried Plasma Fed to Weanling Pigs.

[31] Zimmerman D. (1996). The Duration of Carry-Over Growth Response to Intestinal Hydrolysate Fed to Weanling Pigs.

[32] Frikha M., Mohiti-Asli M., Chetrit C., Mateos G.G. (2014). Hydrolyzed porcine mucosa in broiler diets: Effects on growth performance, nutrient retention, and histomorphology of the small intestine. Poult. Sci..

[33] Opheim M., Sterten H., Øverland M., Kjos N.P. (2016). Atlantic salmon (Salmo salar) protein hydrolysate–Effect on growth performance and intestinal morphometry in broiler chickens. Livest. Sci..

[34] Bui H.T.D., Khosravi S., Fournier V., Herault M., Lee K.-J. (2014). Growth performance, feed utilization, innate immunity, digestibility and disease resistance of juvenile red seabream (Pagrus major) fed diets supplemented with protein hydrolysates. Aquaculture.

[35] Hevrøy E., Espe M., Waagbø R., Sandnes K., Ruud M., Hemre G.I. (2005). Nutrient utilization in Atlantic salmon (Salmo salar L.) fed increased levels of fish protein hydrolysate during a period of fast growth. Aquac. Nutr..

[36] Refstie S., Olli J.J., Standal H.J.A. (2004). Feed intake, growth, and protein utilisation by post-smolt Atlantic salmon (Salmo salar) in response to graded levels of fish protein hydrolysate in the diet. Aquaculture.

[37] Parvez S., Malik K.A., Ah Kang S., Kim H.Y. (2006). Probiotics and their fermented food products are beneficial for health. J. Appl. Microbiol..

[38] Ambalam P., Prajapati J., Dave J., Nair B.M., Ljungh Å., Vyas B.J.M. (2009). Isolation and characterization of antimicrobial proteins produced by a potential probiotic strain of human Lactobacillus rhamnosus 231 and its effect on selected human pathogens and food spoilage organisms. Microb. Ecol. Health Dis..

[39] Pithva S., Ambalam P., Dave J.M., Vyas B.R. (2012). Potential of Probiotic Lactobacillus Strains as Food Additives.

[40] Lu R., Fasano S., Madayiputhiya N., Morin N.P., Nataro J., Fasano A. (2009). Isolation, identification, and characterization of small bioactive peptides from Lactobacillus GG conditional media that exert both anti-Gram-negative and Gram-positive bactericidal activity. J. Pediatr. Gastroenterol. Nutr..

[41] Council N.R. (2012). Nutrient Requirements of Swine.

[42] Kozich J.J., Westcott S.L., Baxter N.T., Highlander S.K., Schloss P.D. (2013). Development of a dual-index sequencing strategy and curation pipeline for analyzing amplicon sequence data on the MiSeq Illumina sequencing platform. Appl. Environ. Microbiol..

[43] Wang X., Tsai T., Deng F., Wei X., Chai J., Knapp J., Apple J., Maxwell C.V., Lee J.A., Li Y. (2019). Longitudinal investigation of the swine gut microbiome from birth to market reveals stage and growth performance associated bacteria. Microbiome.

[44] Schloss P.D., Westcott S.L., Ryabin T., Hall J.R., Hartmann M., Hollister E.B., Lesniewski R.A., Oakley B.B., Parks D.H., Robinson C.J. (2009). Introducing mothur: Open-source, platform-independent, community-supported software for describing and comparing microbial communities. Appl. Environ. Microbiol..

[45] The MIXED Procedure (2018). SAS/STAT® 15.1 User’s Guide.

[46] Wicklin R. (2010). Statistical Programming with SAS/IML Software.

[47] Xiao Y., Kong F., Xiang Y., Zhou W., Wang J., Yang H., Zhang G., Zhao J. (2018). Comparative biogeography of the gut microbiome between Jinhua and Landrace pigs. Sci. Rep..

[48] Coates M.E., Fuller R., Harrison G., Lev M., Suffolk S.F. (1963). A comparision of the growth of chicks in the Gustafsson germ-free apparatus and in a conventional environment, with and without dietary supplements of penicillin. Br. J. Nutr..

[49] Xie Y., He Y., Irwin P.L., Jin T., Shi X. (2011). Antibacterial activity and mechanism of action of zinc oxide nanoparticles against Campylobacter jejuni. Appl. Environ. Microbiol..

[50] Feng Y., Min L., Zhang W., Liu J., Hou Z., Chu M., Li L., Shen W., Zhao Y., Zhang H. (2017). Zinc oxide nanoparticles influence microflora in ileal digesta and correlate well with blood metabolites. Front. Microbiol..

[51] Imtiaz F., Shafique K., Mirza S.S., Ayoob Z., Vart P., Rao S. (2012). Neutrophil lymphocyte ratio as a measure of systemic inflammation in prevalent chronic diseases in Asian population. Int. Arch. Med..

[52] Rosales C., Demaurex N., Lowell C.A., Uribe-Querol E. (2016). Neutrophils: Their role in innate and adaptive immunity. J. Immunol. Res..

[53] Azab B., Zaher M., Weiserbs K.F., Torbey E., Lacossiere K., Gaddam S., Gobunsuy R., Jadonath S., Baldari D., McCord J. (2010). Usefulness of neutrophil to lymphocyte ratio in predicting short-and long-term mortality after non–ST-elevation myocardial infarction. Am. J. Cardiol..

[54] Boissier R., Campagna J., Branger N., Karsenty G., Lechevallier E. (2017). The prognostic value of the neutrophil-lymphocyte ratio in renal oncology: A review. Urologic Oncology: Seminars and Original Investigations.

[55] DiGangi C. (2016). Neutrophil-lymphocyte ratio: Predicting cardiovascular and renal complications in patients with diabetes. J. Am. Assoc. Nurse Pract..

[56] Gomez D., Farid S., Malik H., Young A., Toogood G., Lodge J., Prasad K.R. (2008). Preoperative neutrophil-to-lymphocyte ratio as a prognostic predictor after curative resection for hepatocellular carcinoma. World J. Surg..

[57] Melo M.C.A., Garcia R.F., de Araújo C.F.C., Abreu R.L.C., de Bruin P.F.C., de Bruin V.M.S. (2019). Clinical significance of neutrophil-lymphocyte and platelet-lymphocyte ratios in bipolar patients: An 18-month prospective study. Psychiatry Res..

[58] Xu L., Pan Q., Lin R. (2016). Prevalence rate and influencing factors of preoperative anxiety and depression in gastric cancer patients in China: Preliminary study. J. Int. Med. Res..

[59] Al-Hussain F., Alfallaj M.M., Alahmari A.N., Almazyad A.N., Alsaeed T.K., Abdurrahman A.A., Murtaza G., Bashir S. (2017). Relationship between neutrophil-to-lymphocyte ratio and stress in multiple sclerosis patients. J. Clin. Diagn. Res. JCDR.

[60] Akimova E., Lanzenberger R., Kasper S. (2009). The serotonin-1A receptor in anxiety disorders. Biol. Psychiatry.

[61] Cryan J.F., Dinan T.G. (2012). Mind-altering microorganisms: The impact of the gut microbiota on brain and behaviour. Nat. Rev. Neurosci..

[62] Tsai T., Sales M.A., Kim H., Erf G.F., Vo N., Carbonero F., Van Der Merwe M., Kegley E.A., Buddington R.K., Wang X. (2018). Isolated rearing at lactation increases gut microbial diversity and post-weaning performance in pigs. Front. Microbiol..

[63] Li N., Huang S., Jiang L., Wang W., Li T., Zuo B., Li Z., Wang J. (2018). Differences in the gut microbiota establishment and metabolome characteristics between low-and normal-birth-weight piglets during early life. Front. Microbiol..

[64] Tappenden K.A., Deutsch A.S. (2007). The physiological relevance of the intestinal microbiota-contributions to human health. J. Am. Coll. Nutr..

[65] Kaci G., Goudercourt D., Dennin V., Pot B., Doré J., Ehrlich S.D., Renault P., Blottière H.M., Daniel C., Delorme C. (2014). Anti-inflammatory properties of Streptococcus salivarius, a commensal bacterium of the oral cavity and digestive tract. Appl. Environ. Microbiol..

[66] Mel’nikova E., Koroleva N. (1975). Capacity of Lb. bulgaricus and Str. thermophilus starter to produce antibiotic substances. Dairy Sci. Abstr..

[67] Akpinar A., Yerlikaya O., Kiliccedil S. (2011). Antimicrobial activity and antibiotic resistance of Lactobacillus delbrueckii ssp. bulgaricus and Streptococcus thermophilus strains isolated from Turkish homemade yoghurts. Afr. J. Microbiol. Res..

[68] Kasraei S., Sami L., Hendi S., AliKhani M.-Y., Rezaei-Soufi L., Khamverdi Z. (2014). Antibacterial properties of composite resins incorporating silver and zinc oxide nanoparticles on *Streptococcus mutans* and *Lactobacillus*. Restor. Dent. Endod..

[69] Starke I.C., Pieper R., Neumann K., Zentek J., Vahjen W. (2014). The impact of high dietary zinc oxide on the development of the intestinal microbiota in weaned piglets. FEMS Microbiol. Ecol..

[70] Lin J., Hunkapiller A.A., Layton A.C., Chang Y.-J., Robbins K.R. (2013). Response of intestinal microbiota to antibiotic growth promoters in chickens. Foodborne Pathog. Dis..

[71] Duncan S.H., Louis P., Flint H.J. (2004). Lactate-utilizing bacteria, isolated from human feces, that produce butyrate as a major fermentation product. Appl. Environ. Microbiol..

[72] Ushida K., Kishimoto A., Piao S.J., Itoh M., Shiga A., Nakanishi N., Tsukahara T.J. (2009). An epidemiological survey on pigs showing symptoms of infectious enteric diseases and dyspepsia in Japan. Anim. Sci. J..

[73] Hashizume K., Tsukahara T., Yamada K., Koyama H., Ushida K. (2003). Megasphaera elsdenii JCM1772T normalizes hyperlactate production in the large intestine of fructooligosaccharide-fed rats by stimulating butyrate production. J. Nutr..

[74] Sakata T. (1997). Influence of short chain fatty acids on intestinal growth and functions. Dietary Fiber in Health and Disease.

[75] Tsukahara T., Iwasaki Y., Nakayama K., Ushida K. (2003). Stimulation of butyrate production in the large intestine of weaning piglets by dietary fructooligosaccharides and its influence on the histological variables of the large intestinal mucosa. J. Nutr. Sci. Vitaminol..

[76] Shimotoyodome A., Meguro S., Hase T., Tokimitsu I., Sakata T.J.C.B., Molecular P.P.A., Physiology I. (2000). Short chain fatty acids but not lactate or succinate stimulate mucus release in the rat colon. Comp. Biochem. Physiol. Part A Mol. Integr. Physiol..

[77] Holtug K., Rasmussen H.S., Mortensen P.B. (1992). An in vitro study of short-chain fatty acid concentrations, production and absorption in pig (Sus scrofa) colon. Comp. Biochem. Physiol..

[78] Roediger W., Moore A. (1981). Effect of short-chain fatty acid on sodium absorption in isolated human colon perfused through the vascular bed. Dig. Dis. Sci..

[79] Starke I., Pieper R., Vahjen W., Zentek J. (2014). The impact of dietary Zinc Oxide on the bacterial diversity of the small intestinal microbiota of weaned piglets. J. Vet. Sci. Technol..

[80] Hillman E.T., Lu H., Yao T., Nakatsu C.H. (2017). Microbial ecology along the gastrointestinal tract. Microbes Environ..

