# Supplementary material for: ZnO Modulates Swine Gut Microbiota and Improves Growth Performance of Nursery Pigs When Combined with Peptide Cocktail
Source: Microorganisms. 2020 Jan 21;8(2):146. doi: 10.3390/microorganisms8020146 (PMC7074828; doi:10.3390/microorganisms8020146)
Supplement: Supplementary file 1 [file microorganisms-08-00146-s001.pdf]

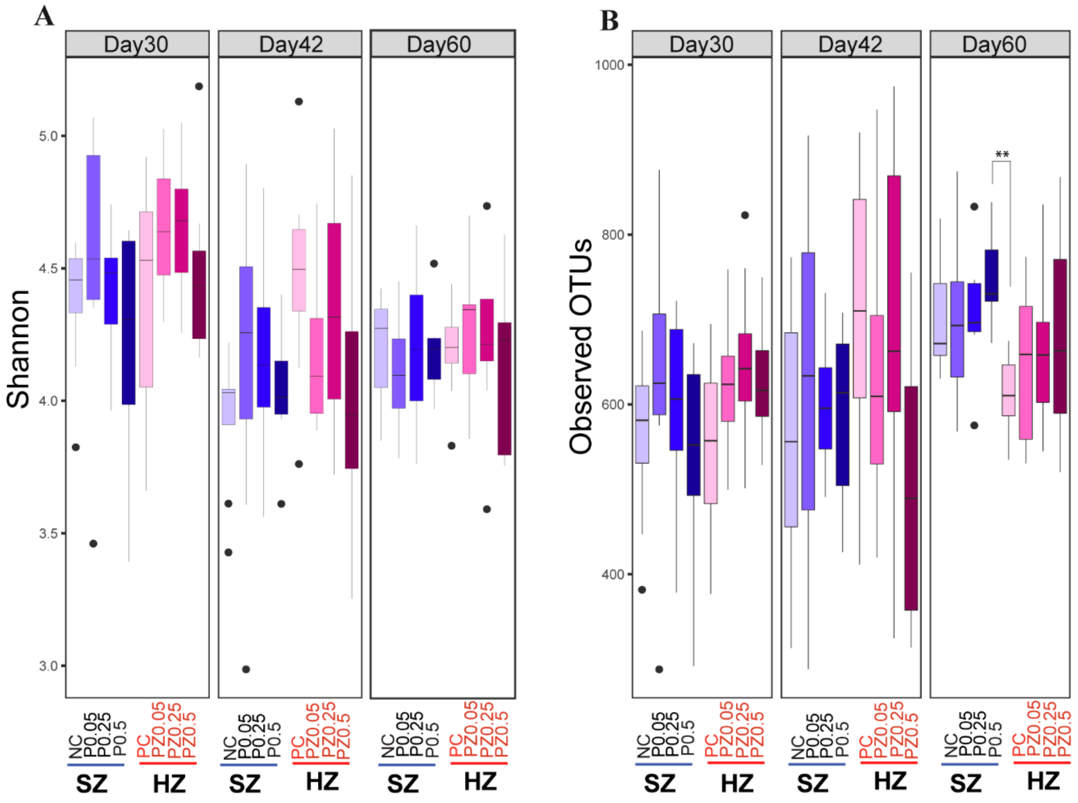

Figure S1. Alpha diversity measures for different dietary supplements at three time points. Common indicators such as Shannon (**A**) and Observed OTUs (**B**) were used to measure bacterial diversity in all subgroups. Comparisons between subgroups were assessed by pairwise Wilcoxon Ranks Sum test with post hoc Bonferroni test.

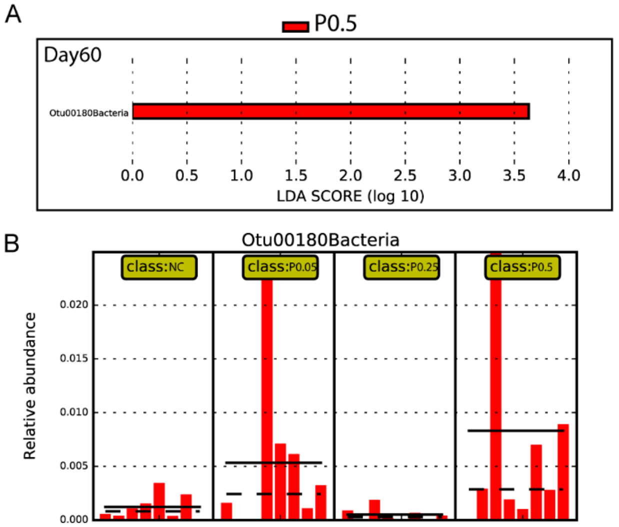

Figure S2. LEfSe analyses of gut bacteria in response to different levels of peptide in SZ group. (**A**) LEfSe identified significantly different bacterial taxa (at OTU level) among subgroups. OTUs in this graph were statistically significant ($p < 0.05$) and had LDA Score >3.5, which was considered a significant effect size. (**B**) Relative abundance of Otu180 in pig rectum. The means and medians are shown as solid and dashed lines in each subgroup. Each column represents one animal.

Table S1. Dietary treatments.

Standard Zinc	NC	nutrient deficient diet (removed fishmeal in phase2; reduced soybean meal in phase 3)
	P0.05	NC+0.05% Peptide
	P0.25	NC+0.25% Peptide
	P0.5	NC+0.5% Peptide
High Zinc	PC	nutrient sufficient diet+ZnO
	PZ0.05	NC+0.05% Peptide +ZnO
	PZ0.25	NC+0.25% Peptide +ZnO
	PZ0.5	NC+0.5% Peptide +ZnO

Table S2. Peptiva nutrient composition (as fed).

**Nutrient Composition**	**Percentage**
Crude protein:	51.25
Lysine	2.72
Methionine	0.73
Phenylalanine	1.72
Threonine	1.59
Tyrosine	1.16
Valine	1.93
Iso-leucine	1.58
Leucine	2.99
Crude fat	6.34
Fiber	5.38
Ash	11.31
Moisture	5.97
Other	19.75

Table S3. Diet Ingredient.

	**Phase 1**	**Phase 2**		**Phase 3**	
		**PC**	**NC**	**PC**	**NC**
Ingredients, %					
Corn	39.77	56.38	59.06	50.29	54.79
Soybean meal	22.5	27.60	27.60	28.20	23.96
Corn DDGS	0	5.00	5.00	15.00	15.00
Monocalcium P	0.65	0.54	0.94	0.40	0.45
Limestone	0.45	0.93	1.16	1.10	1.11
Salt	0.25	0.50	0.50	0.50	0.50
L-Lysine	0.21	0.42	0.42	0.47	0.47
DL-Methionine	0.165	0.18	0.18	0.15	0.15
L-Threonine	0.049	0.13	0.13	0.11	0.11
L-Tryptophan	0.0275	0.04	0.04	0.03	0.03
ZnO	0	0.32		0.32	
Plasma (AP-920)	3	1.50	1.50		
Fish Meal, Menhaden	6	3.00			
Milk, Whey Powder	20				
L-Valine	0	0.04	0.04		
Milk, Lactose	3.5				
Other*	3.43	3.43	3.43	3.43	3.43
Total	100	100	100	100	100
Calculate					

ME, kcal/kg	3487	3437	3427	3426	3439
CP (%)	22.81	23.0	21.3	22.4	20.8
SID Lysine (%)	1.46	1.42	1.29	1.28	1.18
Total P (%)	0.77	0.60	0.60	0.51	0.50
Available P (%)	0.55	0.35	0.35	0.25	0.25
Aval. P (%) with phytase	0.58	0.38	0.38	0.28	0.28
Ca (%)	0.85	0.76	0.76	0.65	0.65
SID M+C:Lys	58.07	58.0	59.6	58.1	60.1
SID Thr:Lys	60.09	60.0	61.2	60.0	60.4
SID Trp:Lys	20.05	19.1	19.7	19.0	18.7
SID Ile:Lys	58.98	56.7	57.4	60.2	59.5
SID Val:Lys	66.92	67.0	68.0	67.0	67.0
SID Leu:Lys	118.48	121.1	125.3	134.5	138.0

Others: Poultry fat (3%), trace mineral premix (0.15%), Vitamin premix (0.25%), antioxidant (0.03%), and phytase (0.003%) were added to all diets.

All pigs were fed common phase1 diet and then experimental phase2 and phase3 diets were introduced. Peptide was added on negative control diet at concentration of 0.05%, 0.25% and 0.5% to achieve P0.05, P0.25, and P0.5, while additional Zn as ZnO together with 0.05%, 0.25% and 0.5% of peptide was added to form PZ0.05, PZ0.25, and PZ0.5. Phase1: d 21-30; Phase2: d31-42; Phase3: d43-60.

Table S4. Effect of Peptide cocktail on BW and ADG in nursery pigs fed diets with or without ZnO (least square means ± SE) [1].

	PC	NC	P0.05	P0.25	P0.5	PZ0.05	PZ0.25	PZ0.5	P -Value
**ADG, kg/d**									
NP2	0.274±0.018^{ab}	0.253±0.017^{ab}	0.235±0.017^{ab}	0.224±0.017^{a}	0.272±0.017^{ab}	0.234±0.018^{ab}	0.307±0.017^{bc}	0.360±0.017^{c}	<0.0001
NP3	0.609±0.025^{b}	0.539±0.024^{ab}	0.562±0.024^{ab}	0.505±0.024^{a}	0.543±0.024^{ab}	0.585±0.025^{b}	0.604±0.024^{b}	0.592±0.024^{b}	0.0012
NP2-3	0.500±0.018^{cd}	0.441±0.018^{ab}	0.453±0.018^{abc}	0.407±0.018^{a}	0.452±0.018^{abc}	0.477±0.018^{bcd}	0.502±0.018^{cd}	0.514±0.018^{d}	<0.0001
**BW, kg**									
NP1	5.60±0.45	5.73±0.45	5.65±0.45	5.64±0.45	5.69±0.45	5.44±0.45	5.63±0.45	5.60±0.45	0.1771
NP2	8.34±0.51^{ab}	8.32±0.51^{a}	8.08±0.51^{a}	7.88±0.51^{a}	8.41±0.51^{ab}	7.77±0.51^{a}	8.70±0.51^{ab}	9.20±0.51^{b}	0.0006
NP3	20.22±0.93^{bc}	18.57±0.92^{ab}	18.90±0.92^{abc}	17.45±0.92^{a}	18.83±0.92^{abc}	19.38±0.94^{bc}	20.21±0.92^{bc}	20.51±0.92^{c}	<0.0001

Notes: Pigs were fed common phase 1 diet for 10 days and then switched to experimental diets for nursery phase (NP) 2 and 3. Least square means results are presented. Data with different superscripts in the same row indicate significant different ($p < 0.05$). ADG: average daily gain; BW: body weight (recorded on the end of each phase). NP1: d 21-30; NP2: d31-42; NP3: d43-60. [1] ADG (NP1) and feed efficiency (NP1) were used as covariant for BW and ADG analysis.

Table S5. Effect of Peptide cocktail on ADFI and G:F in nursery pigs fed diets with or without ZnO(least square means ± SE)[1].

	PC	NC	P0.05	P0.25	P0.5	PZ0.05	PZ0.25	PZ0.5	P - Value
**ADFI, kg/d**									
NP2	0.315±0.018	0.310±0.017	0.292±0.017	0.301±0.017	0.310±0.017	0.299±0.019	0.345±0.017	0.336±0.017	0.3173
NP3	0.682±0.037	0.643±0.037	0.719±0.037	0.626±0.036	0.663±0.037	0.724±0.041	0.746±0.036	0.770±0.037	0.0288
NP2-3	0.555±0.027	0.528±0.027	0.572±0.027	0.514±0.027	0.541±0.027	0.577±0.030	0.608±0.027	0.620±0.027	0.0338
**G:F**									
NP2	0.883±0.048^{ab}	0.797±0.046^{a}	0.772±0.046^{a}	0.749±0.046^{a}	0.893±0.047^{ab}	0.783±0.049^{a}	0.890±0.046^{ab}	1.056±0.047^{b}	0.0011
NP3	0.864±0.028	0.840±0.027	0.791±0.027	0.808±0.027	0.827±0.027	0.798±0.029	0.818±0.027	0.772±0.027	0.3367

NP2-3	0.867±0.024	0.832±0.024	0.786±0.024	0.796±0.024	0.837±0.024	0.796±0.025	0.833±0.024	0.827±0.024	0.2666

Notes: Pigs were fed common phase1 diet for 10 days and then switched to experimental diets for nursery phase (NP) 2 and 3. Least square means results are presented. Data with different superscripts in the same row indicate significant differences ($p < 0.05$). ADFI: average daily feed intake; G:F: feed efficiency. NP1: d 21-30; NP2: d31-42; NP3: d43-60. [1]ADFI (NP1) and feed efficiency (NP1) were used as a covariant for ADFI analysis, while feed efficiency was used as covariant for feed efficiency analysis.

Table S6. Contrast result of BW and ADG (covariance included).

	No Zinc		Zinc			
	Linear Pep	Quad. Pep	Linear Pep	Quad. Pep	Linear Pep * Zinc	Quad. Pep * Zinc
ADG, kg/d						
NP2	0.1162	0.1969	<.0001	0.4313	0.0182	0.1473
NP3	0.5351	0.0211	0.8307	0.4554	0.5512	0.0335
NP2-3	0.8813	0.0053	0.0638	0.5493	0.1948	0.0174
BW, kg						
NP1	0.6229	0.7200	0.1167	0.1322	0.3998	0.1856
NP2	0.2090	0.1514	<.0001	0.2441	0.0131	0.0711
NP3	0.9527	0.0050	0.0721	0.5078	0.1880	0.0150

Notes: Pigs were fed common phase 1 diet for 10 days and then switched to experimental diets for nursery phase (NP) 2 and 3. ADG: average daily gain; BW: body weight (recorded on the end of each phase). NP1: d 21-30; NP2: d31-42; NP3: d43-60.

Table S7. Analysis of similarity (ANOSIM) was used to study swine gut microbiome dissimilarities between the different levels of peptide within SZ or HZ group based on the Braycurtis or Jaccard distances.

Braycurtis

	Day30 (starting point)		Day42		Day60	
	R	P	R	P	R	P
NC vs P0.05	-0.04	0.67	0.00	0.48	-0.09	0.84
NC vs P0.25	0.03	0.25	-0.02	0.54	0.03	0.34
NC vs P0.5	0.03	0.30	-0.07	0.74	0.01	0.38
PC vs PZ0.05	0.03	0.28	-0.04	0.73	0.01	0.40
PC vs PZ0.25	0.05	0.28	-0.13	0.99	0.04	0.22
PC vs PZ0.5	-0.05	0.76	-0.03	0.63	0.03	0.28

Jaccard

	Day30 (starting point)		Day42		Day60	
	R	P	R	P	R	P
NC vs P0.05	-0.03	0.67	-0.02	0.53	0.00	0.45
NC vs P0.25	-0.05	0.76	0.09	0.06	0.03	0.26
NC vs P0.5	-0.02	0.56	-0.07	0.87	-0.03	0.69
PC vs PZ0.05	0.02	0.29	-0.04	0.66	-0.02	0.56
PC vs PZ0.25	0.02	0.36	-0.11	1.00	0.00	0.50
PC vs PZ0.5	0.00	0.38	0.07	0.13	0.01	0.41
